# Time-series alignment by non-negative multiple generalized canonical correlation analysis

**DOI:** 10.1186/1471-2105-8-S10-S4

**Published:** 2007-12-21

**Authors:** Bernd Fischer, Volker Roth, Joachim M Buhmann

**Affiliations:** 1 Institute of Computational Science, ETH Zurich, Switzerland; 2 Competence Center of Systems Physiology and Metabolic Diseases, ETH Zurich, Switzerland

## Abstract

**Background:**

Quantitative analysis of differential protein expressions requires to align temporal elution measurements from liquid chromatography coupled to mass spectrometry (LC/MS). We propose *multiple Canonical Correlation Analysis *(mCCA) as a method to align the non-linearly distorted time scales of repeated LC/MS experiments in a robust way.

**Results:**

Multiple canonical correlation analysis is able to map several time series to a consensus time scale. The alignment function is learned in a supervised fashion. We compare our approach with previously published methods for aligning mass spectrometry data on a large proteomics dataset. The proposed method significantly increases the number of proteins that are identified as being differentially expressed in different biological samples.

**Conclusion:**

Jointly aligning multiple liquid chromatography/mass spectrometry samples by mCCA substantially increases the detection rate of potential bio-markers which significantly improves the interpretability of LC/MS data.

## Background

Liquid chromatography coupled to mass spectrometry (LC/MS) has emerged as the technology of choice for the quantitative analysis of proteins over the last decade. Technically, the liquid chromatography process separates solute molecules of a multi component chemical mixture which are measured by LC detectors as a time series of solute mass. A major problem when comparing two biological samples measured with LC/MS is a non-linear deformation of the time scale between two experiments. A LC/MS device generates mass peaks along the time axis. When two mass spectrometry experiments are aligned, it is our goal to generate matching hypotheses for as many peaks as possible between the two runs, while ensuring that most of these hypotheses are correct.

One of the standard methods for aligning mass spectrometry experiments is called *correlation optimized warping *(COW) [1], where piece-wise linear functions are fitted to align pairs of time series. A hidden Markov model [2] was proposed to align the mass spectrometry data as well as acoustic time series. In [3] the model was extended to use more than one *m/z *bin for aligning. Tibshirani [4] proposed hierarchical clustering for aligning. Kirchner et al. [5] resorted to robust point matching as developed in medical image analysis. All these previous methods did not utilize the information of identified peptides that are available in tandem mass spectrometry. In our previous work on LC/MS alignment [6] we addressed this problem by way of a semi-supervised nonlinear ridge regression model that maps one time scale onto the other. While this model has been demonstrated to outperform other approaches, it still suffers from two methodological shortcomings: (i) the regression approach is non-symmetric. By mapping the first experiment onto the second one can yield results different from mapping the second onto the first; (ii) the method is limited to aligning only *pairs *of time series, whereas in many experiments we have access to more than two replica. In this paper we will extend the ideas proposed in [6] by a *symmetric *approach based on *canonical correlation analysis*. mCCA is capable of aligning *multiple *time series and, thus, effectively benefits from an enlarged training set.

### Biological motivation

In quantitative proteomics one is interested in classifying a protein sample (e.g. blood plasma) according to some phenotypes, e.g. distinguishing between cancer and non-cancer on the basis of a blood plasma sample. Moreover, in many applications it is of particular interest to identify those proteins that are *relevant *for the discrimination between different biological conditions. In bottom-up proteomics, the proteins are first digested by an enzyme into smaller sized pieces, called peptides. Let $pa_{i}^{\left(1\right)}$ and $pa_{i}^{\left(2\right)}$ be the (measured) amount of ions of peptide *i *in sample 1 and 2. According to [6] the differential protein expression estimate ${\hat{\delta }}_{p}$ can be estimated as

$${\hat{\delta }}_{p}=\frac{1}{n}\sum_{i=1}^{n}\left(\log\left(pa_{i}^{\left(1\right)}\right)-\log\left(pa_{i}^{\left(2\right)}\right)\right)$$

**Figure 1:**
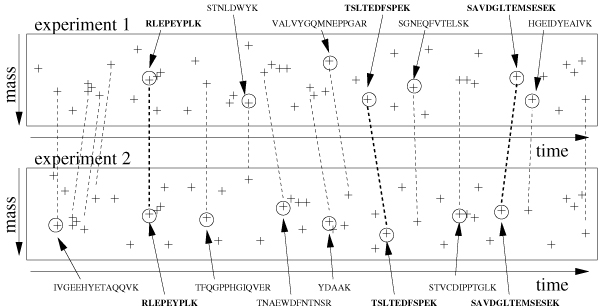
A sketch of an LC/MS alignment. The crosses depict detected peaks, the circles depict identified peaks.

The above differential protein expression estimate is the mean of the log-ratios of peptide expressions over all peptides that correspond to a particular protein. Due to unknown ionization efficiency and digestion rate only the differential protein expression value can be reliably estimated [6,7]; absolute expression level cannot be robustly measured in precision experiments. The basis for estimating differential protein expressions is a large set of peptides that are measured in both samples. This work primarily addresses the issue to reliably find correspondences between peptide measurements in several replicated samples. Liquid chromatography/mass spectrometry (LC/MS) allows us to measure the amount of peptide ions. Figure 1 schematically depicts two LC/MS experiments. The time corresponds to the retention time when the peptide ion elutes from the liquid chromatography column. Ions with the same peptide structure will elute within a small time window. After some preprocessing (see [6]) one gets a list of peaks within the two dimensional image with a mass/charge coordinate and a time coordinate. Each cross in Figure 1 depicts a peptide (with a certain charge state). In addition, the amount of peptide ions *pa*_*i *_is measured by the peak intensities.

For some peaks we have access to the underlying peptide sequence. The machine randomly selects a small number of peaks (typically 3) among the largest peaks of the MS spectrum. Peptide ions within a small mass/charge window are selected and stabilized in an ion trap. The selected peptide ions are further fragmented by a collision with a noble gas. A tandem mass spectrum (MS/MS) is acquired from these fragment ions. These peptide sequences are estimated based on MS/MS data, which contain a dissociation pattern of the peptide ions (see [8,9] for details about peptide identification). In Figure 1 peaks with known peptide sequence are marked with a circle. In practice, this subset of identified peaks appears like a random selection (since the peptide masses for subsequent MS/MS spectra acquisition are selected randomly) and, consequently, the overlap of jointly identified peptides between replicated experiments is small. Since the measurement process is rather time consuming, the LC/MS machine selects only a small number of peaks for MS/MS scans and further identification.

When an experiment is repeated several times (technical replicates), one often observes that the mass axis is usually conserved very well, but the time axis shows substantial non-linear deformations. Since the mass axis is expected to contain only negligible errors and to keep the notation simple, we will not explicitly mention the mass measurement in the sequel.

In summary we have two different sets of objects for every experiment:

1. A large list of peaks at various time points *without *knowledge about the underlying peptide sequence (typically 2000–3000 peaks).

2. A moderate list of peaks *with *known peptide sequence (typically 100–300 peaks). The overlap of identified peptide sequences between experiments is small (typically 10–40 peaks).

The main idea behind our approach is to increase the number of identified peaks by *aligning *all replicated runs of the experiment. The individual time scales are warped to a *canonical *time scale which allows us to generate matching hypothesis even if the peptide sequences are missing. Focusing on the time measurements, we analyze the correctness of the predicted correspondences in terms of precision-recall statistics.

## Results and discussion

Our model for estimating the time warping function is based on multiple canonical correlation analysis (mCCA). Individual time scales are projected on a canonical scale such that the joint correlation in the projected space is maximized. The projection has to obey the constraint that the warping models the correspondence of monotonic temporal evolutions (i.e. negative time gradients are forbidden). This constraint is satisfied by projecting the time coordinates on a basis of hyperbolic tangent basis functions (generalized CCA) and by including a non-negativity constraint when optimizing the correlation (see the methods section).

As a test set for our aligning method we use 10 different sample pairs from an *Arabidopsis thaliana *cell culture. Each sample pair contains two slightly different biological samples. The different conditions are designed as follows: Given three different samples A, B, and C (in our case consecutive slices of a 1D-gel), the first sample contains a pool of A and B and the second sample contains a pool of B and C. Since the protein abundances on different gel slices are similar to each other, we measure the difference of protein abundance between consecutive gel slices. The two samples (pool A/B and pool B/C) are measured in two single experiments. For every sample 3 technical replicates are available for the analysis. Thus we have 3 LC/MS runs for the pool A/B and additional 3 LC/MS runs for the pool B/C.

The robust mCCA method is used for jointly aligning all 6 experiments available for a pair of samples. The results are then compared to the analysis based on the robust ridge-regression method which has been proposed in [6] as well as thin plate spline fitting [5]. The robust ridge regression technique possibly violates the monotonicity constraint of temporal warping and it has also not been developed for computing multiple alignments. All 6·5/2 pairs of LC/MS experiment are aligned by ridge regression. In addition we compare our method to a pairwise alignment method based on thin-plate splines [5] which is freely available. Instead of implicitly estimating the point correspondences, we fixed the given correspondences. All three methods produce a (possible empty) list of peaks where every peak is either identified by MS/MS or by prediction. Contradictions are resolved by majority vote.

### Validation of peak matching with known peptide sequence

The three methods are compared by 10-fold cross validation using the known labels of the peaks. All peptides that are identified in one of the 6 LC/MS experiments, are partitioned in 10 folds. Ridge regression, thin plate splines and multiple CCA have then been trained on 9 folds and the agreement on peptide sequence is then tested on the remaining fold. To measure the agreement, the number of peptides that are assigned to the same peak, are summed over all test peptides that are identified jointly in one pair of experiments and over all 6·5/2 pairs of experiments. We like to emphasize here, that even if a test peptide is compared in a pair of alignments, this test peptide did not appear in the training set of any other experiment.

The following three cases are considered in the evaluation:

1. **no match. **The peak is not assigned to any peak in the second time series.

2. **correct. **The peak is assigned to the peak with the same label.

3. **wrong. **The peak is assigned to the peak with another label.

From these categories we then compute *precision *and *recall *values as follows:

$$\begin{array}{cccc} \left(\text{recall}\right) & rec & = & \frac{\#correct+\#wrong}{\#correct+\#wrong+\#nomatch} \end{array}$$

$$\begin{array}{cccc} \left(\text{precision}\right) & prec & = & \frac{\#correct}{\#correct+\#wrong} \end{array}$$

**Figure 2:**
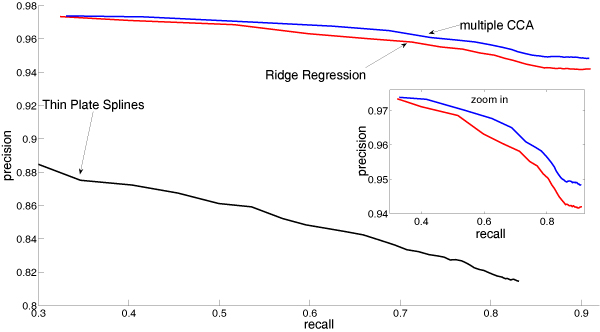
Precision-Recall-Curve for the labeled peaks. "Ridge-regression" refers to the method proposed in [6].

The recall is defined as the number of peaks that are assigned to a peak with the same peptide sequence relative to the total number of (labeled) peaks. Each labeled peak can either be assigned to a peak correctly, to a wrong peak or to no peak. The precision value is the number of peaks that are assigned to the correct peak among the set of peaks that could be assigned to any other peak (excluding the peaks that could not be assigned: $f_{k,l}(t_{j}^{k})=\emptyset$ in Equation 8). In Figure 2 the precision-recall curves are plotted. We conclude that robust multiple CCA outperforms robust ridge regression consistently by more than five percent in recall for a given precision value. The thin plate splines perform much worse than the robust mCC and robust ridge regression. The runtime for the different methods for the whole dataset are 33 sec. for robust ridge regression, 6 min. 28 sec. for the robust mCCA and 19 hours 45 min. for the thin plate spline implementation by Kirchner [5]. The runtime for the thin plate splines are only for one parameter setting whereas the runtime for ridge regression and CCA includes a model selection over ten different parameter (polynomial degree and *σ *of hyperbolic tangent functions). There possibly exists a better parameter choice for the thin plate splines, but due to the enormous runtime, we could only select the parameters on a small sized example.

### Validation of differential protein expression values

In practical applications, the most important quality criterion for alignment methods of this kind is the number of proteins that are detected as significantly over-/underexpressed. In order to estimate this number, all six experiments are pair-wise aligned and log-peptide abundance ratios are estimated for all jointly identified peptides. Equation 1 suggests to calculate the mean log-peptide abundance ratio averaged over all peptides which originate from a particular protein. If this average log ratio deviates with t-test significance level *α *from zero then we declare this protein as strongly under- or overexpressed between the two conditions. The t-test with significance level *α *provides us with a list of differently expressed proteins. This test can be applied to two samples measured (i) under different biological conditions or (ii) as technical replicates. If the percentage of proteins detected as differentially abundant in different biological conditions *p*_diff _substantially exceeds the percentage of proteins detected as differentially abundant in replicates *p*_rep_, then we can conclude that the difference in these biological conditions significantly influences the proteome. The reader should note that one should compare against biological replicates, to detect significant biomarkers. Unfortunately for our analysis, no biological replicates are available. But the technical replicates are still sufficient to show that the underlying method is able to detect differences in biological samples from different experimental conditions.

A detection rate *p*_rep _= *α *for a t-test with significance level *α *can be expected due to statistical fluctuations. We observed for our experiments that *p*_rep _usually varies between 4% and 5% for *α *= 3% which is acceptable. The ratio *p*_rep_/*p*_diff _gives us now an estimate of the false discovery rate in the set of proteins detected as significantly different abundant. Changes in the significance level *α *controls the false discovery rate. The number of true-positive proteins are now estimated by the formula #positive·(1 - false discovery rate).

**Figure 3:**
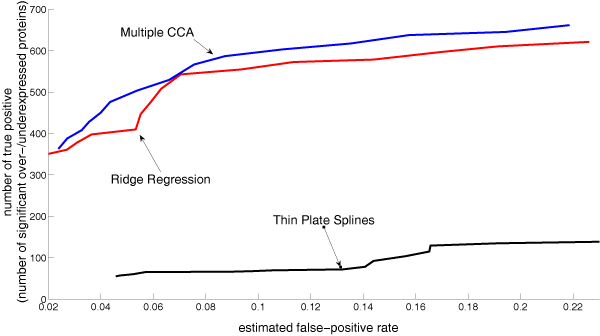
Number of proteins classified as significantly over-/underexpressed as function of the estimated false discovery rate.

Figure 3 shows the number of significantly different abundant proteins as a function of the false discovery rate. Multiple CCA clearly outperforms ridge regression in the number of estimated differential protein abundance levels at the same false discovery rate.

**Figure 4:**
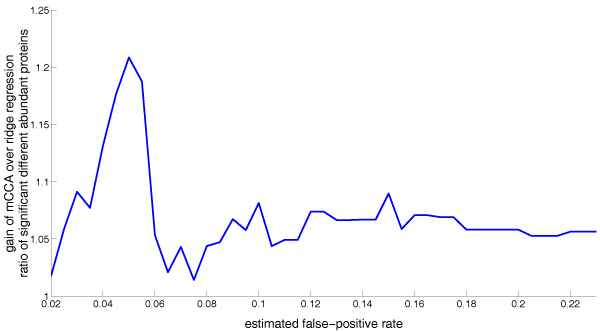
Ratio of the number of proteins classified as significantly over-/underexpressed as function of the estimated false discovery rate. The ratio is between multiple CCA and pair-wise ridge regression.

To compare the sensitivities of the alignment methods, we compare the number of differently abundant proteins detected by multiple CCA with the detections by ridge regression. The ratio of these two detection rates is shown as a function of the false discovery rate in Figure 4. The gain by multiple CCA is between 2% and 22% for different false discovery rates. The choice of a suitable false discovery rate depends on the proteomics application. In a biomarker discovery scenario we are interested in a fairly small false discovery rate to reduce the amount of work for experimental validation. In high throughput screening scenarios, bio-scientists are interested to find *potential *bio-markers that are further investigated by an additional subsequent analysis, and, therefore, they can accept more false discovery detections.

## Conclusion

In this paper we are concerned with one of the critical steps in the data analysis of quantitative differential proteomics experiments. If an experiment with a liquid chromatography unit is repeated, one typically observes a non-linear deformation of the time scales.

A novel technique for aligning such time scales is proposed where the alignment method is based on generalized canonical correlation analysis with a built-in non-negativity constraint. Two severe problems of previous approaches are solved with the novel technique: (i) non-symmetry of the time prediction function and (ii) a potential violation of the monotonicity constraint which is inherent in temporal alignments. On a large proteomics dataset we demonstrate that jointly aligning multiply replicated experiments increases both precision and recall: the total number of peptide correspondences is increased as well as the quality of these matches is improved by the novel technique. These improvements directly influence estimates of differential protein expression values, because the number of proteins are significantly increased that are detected as differentially abundant in our experiments.

## Methods

### Formal problem description

Assume we have to align *K *different time scales. Each time scale *k *is described by a list of peaks with time coordinates

$$P_{k}=\left\{t_{1}^{\left(k\right)},...,t_{n_{k}}^{\left(k\right)}\right\}$$

Furthermore, a set of known correspondence points between time scales *k *and *l*

$$C_{k,l}=\left\{\left(t_{1}^{\left(k\right)},t_{1}^{\left(l\right)}\right),...,\left(t_{m}^{\left(k\right)},t_{m}^{\left(l\right)}\right)\right\}$$

are provided by data base search. These time points are defined by those peptides that have been identified in both samples. For a peak *p *∈ *P*_*k *_we try to find a corresponding peak *q *∈ *P*_*l *_(if there exists one), which formally amounts to determine a mapping

*f*_*k*,*l *_: *P*_*k *_→ *P*_*l *_∪ {∅}

from the set of peaks *P*_*k *_to the set of peaks *P*_*l *_extended by the symbol ∅. This symbol ∅ represents the case that no corresponding peak can be found on the time scale *l*. Note that Eq. (6) defines a mapping between finite sets of time points.

To find a suitable mapping between the peaks, a continuous transformation between the time scales has to be learned. The function

*g*_*k*,*l *_: ℝ → ℝ

transforms the (continuous) time scale *k *into the (continuous) time scale *l*. Given the time transformation *g*_*k*,*l *_we create the mapping *f*_*k*,*l *_as

$$f_{k,l}\left(t_{j}^{\left(k\right)}\right)=\{\begin{array}{lll} \arg\min_{t_{i}^{\left(l\right)}\in P_{l}}\{d_{ij}\} & \text{if }\exists i:d_{ij}\leq w & \text{where }d_{ij}=\left|t_{i}^{\left(l\right)}-g_{k,l}\left(t_{j}^{\left(k\right)}\right)\right|\\ \emptyset & \text{else}. & \end{array}$$

The peak $t_{j}^{\left(k\right)}$ is mapped to the peak closest to the predicted time on time scale *l *within a window of size *w*. The window size *w *controls the number of accepted correspondences. A smaller window size leads to a lower total number of accepted matches, but to a higher *precision *of the accepted correspondences, i.e. to an increased fraction of correct matches. A continuous time transformation *g*_*k*,*l *_(see the next section for details) is learned and the transformation is evaluated using the mapping function *f*_*k*,*l*_.

### Robust ridge regression

Fischer et al. [6] used robust ridge regression with polynomial basis functions to estimate the time transformation functions *g*_*k*,*l*_. Let $(x_{i},y_{i})=\left(t_{i}^{\left(k\right)},t_{i}^{\left(l\right)}\right)\in C_{k,l}$ be a tuple of known time correspondences between time series *k *and *l*. The time is explicitly transformed to the polynomial basis $\varphi (x_{i})={\left(1,x_{i},x_{i}^{2},...,x_{i}^{d}\right)}^{t}$. Without loss of generality it is assumed that the sample vectors *φ*(*x*_*i*_) have zero mean and unit covariance, otherwise the vectors are normalized accordingly. Robust ridge regression finds the parameter vector *β *that minimizes

$$\sum_{i=1}^{n}L_{c}(\varphi {\left(x_{i}\right)}^{t}\beta -y_{i})+\lambda \beta^{t}\beta ,$$

where *L*(*ξ*) is Huber's loss function [10]

$$L_{c}(\xi )=\{\begin{array}{ll} c\left|\xi \right|-\frac{c^{2}}{c} & ,\text{for }\left|\xi \right|>c\\ \frac{\xi^{2}}{2} & ,\text{for }\left|\xi \right|\leq c. \end{array}$$

However, the use of ridge regression to align time series has two disadvantages:

1. ridge regression is not symmetric in the sense that *g*_*l*,*k *_is not an inverse of *g*_*k*,*l*_: *x *≠ *g*_*l*,*k*_(*g*_*k*,*l*_(*x*));

2. the time transformation function is not necessarily monotonically increasing. It might, thus, violate the monotonicity constraint that is inherent in temporal alignments.

### Canonical correlation analysis

To overcome the above problems of ridge regression we propose to address the alignment task by way of canonical correlation analysis [11]. The two time scales are both mapped on a canonical time axis. Correlation analysis aims to find a linear projection on a canonical time axis such that the correlation between the two random variables is maximized. Again we assume that the sample vectors *φ*(*x*_*i*_) have zero mean and unit covariance. The objective is to find parameter vectors *β*_1 _and *β*_2 _that maximize the correlation between the linear projections *φ*(*x*_*i*_)^*t*^*β*_1 _and *φ*(*x*_*i*_)^*t*^*β*_2_

$$\text{maximize}\frac{\sum_{i=1}^{n}\beta_{1}^{t}\varphi (x_{i})\varphi {\left(y_{i}\right)}^{t}\beta_{2}}{\sqrt{\sum_{i=1}^{n}{\left(\varphi {\left(x_{i}\right)}^{t}\beta_{1}\right)}^{2}\sum_{i=1}^{n}{\left(\varphi {\left(y_{i}\right)}^{t}\beta_{2}\right)}^{2}}}$$

The denominator of Equation 11 can be reformulated as ||*β*_1_||·||*β*_2_||, since the covariance matrices $\Phi_{X}\Phi_{X}^{t}$ and $\Phi_{Y}\Phi_{Y}^{t}$ are normalized to unit covariance before. The problem can be solved directly by a transformation to the eigenvalue problem (see [12])

$$\begin{array}{ccc} \lambda_{max}=\sup\{\beta_{1}^{t}\Phi_{X}\Phi_{Y}^{t}\Phi_{Y}\Phi_{X}^{t}\beta_{1}|\left\|\beta \right\|=1\} & with & \Phi_{x}=(\varphi (x_{1}),...,\varphi (x_{n})). \end{array}$$

The vector *β*_2 _is then obtained by $\beta_{2}=\Phi_{Y}\Phi_{X}^{t}\beta_{1}$. In order to include the time-monotonicity constraint and robustness, however, it is advantageous to use an alternative formalization of the optimization problem, which can be equivalently restated as follows (see [13]):

$$\begin{array}{cc} \text{minimize}\sum_{i=1}^{n}{\left(\varphi {\left(x_{i}\right)}^{t}\beta_{1}-\varphi {\left(y_{i}\right)}^{t}\beta_{2}\right)}^{2} & \text{s}.\text{t}.\;\left\|\beta_{1}\right\|=1,\left\|\beta_{2}\right\|=1. \end{array}$$

The function *g*_*k*_(*x*_*i*_) = *φ*(*x*_*i*_)^*t*^*β*_*k *_denotes the transformation from the time scale *k *to the canonical time scale. Even for this reformulated problem, however, no guarantee is given that the transformation is monotonically increasing. In the case of a non-monotone function it is impossible to find an unambiguous transformation between time scales *k *to *l*, because in general *g*_*k *_is not invertible.

To overcome this problem we propose two changes in the setting of canonical correlation analysis:

1. A set of hyperbolic tangent basis functions is used.

2. A non-negativity constraint on the regression parameters is introduced.

The basis functions are defined as

$$\varphi (x_{i})=\left(\begin{array}{c} \tanh(\sigma (x_{i}-z_{1}))\\ \tanh(\sigma (x_{i}-z_{2}))\\ \vdots \\ \tanh(\sigma (x_{i}-z_{d})) \end{array}\right).$$

The set of vectors *z*_1_,...,*z*_*d *_can either be chosen as the set of vectors *x*_1_,...,*x*_*n *_or as a set of *d *time points equally distributed over the range of the respective time scale. The scaling parameter *σ *controls the smoothness of the estimated alignment function.

If the parameters *β*_1 _and *β*_2 _are non-negative, the function *g*_*k*_(*x*_*i*_) = *φ*(*x*_*i*_)^*t*^*β*_*k *_is monotonically increasing, because it is a linear combination with non-negative coefficients of monotonically increasing (hyperbolic tangent) functions. Instead of hyperbolic tangent functions, any other monotonically increasing and bounded basis functions can be used. The alignment problem can now be defined as non-negative canonical correlation analysis with hyperbolic tangent basis functions. Learning the time warping requires to find the parameter vectors *β*_1 _and *β*_2 _that minimize the objective function

$$\begin{array}{cc} \text{minimize}\sum_{i=1}^{n}{\left(\varphi {\left(x_{i}\right)}^{t}\beta_{1}-\varphi {\left(y_{i}\right)}^{t}\beta_{2}\right)}^{2} & \text{s}.\text{t}.\;\left\|\beta_{1}\right\|=1,\left\|\beta_{2}\right\|=1,\beta_{k,j}\geq 0. \end{array}$$

We solve this problems iteratively by gradient descent. In a first step the objective function is minimized over *β*_1 _while keeping *β*_2 _fixed. Then the length of the vector *β*_1 _is normalized to fulfill the constraint ||*β*_1_|| = 1. In a second step the objective is minimized over *β*_2 _while keeping *β*_1 _fixed, and *β*_2 _is normalized accordingly. In each step of the iteration we have to solve a non-negative least-squares problem for which we use the Lawson-Hanson algorithm [14].

Using the above definition of the correlation problem we can easily extend the correlation coefficient to a *robust *version that is less sensitive to outliers. For that purpose the squared loss is replaced by Huber's robust loss function (Eq. 10)

$$\begin{array}{cc} \text{minimize}\sum_{i=1}^{n}L_{c}(\varphi {\left(x_{i}\right)}^{t}\beta_{1}-\varphi {\left(y_{i}\right)}^{t}\beta_{2}) & \text{s}.\text{t}.\;\left\|\beta_{1}\right\|=1,\left\|\beta_{2}\right\|=1,\beta_{k,j}\geq 0. \end{array}$$

Optimization of the robust CCA functional is similar to its quadratic counterpart (15), but with an additional inner loop that solves the robust non-negative least-squares problems by an iteratively re-weighted non-negative least-squares algorithm.

### Multiple canonical correlation analysis

Sometimes there are only a very few peptides that are measured in both runs. In the case where one has more than two experiments one can benefit from multiple datasets to increase the size of the training dataset. The problem of canonical correlation analysis can be extended to multiple correlation analysis [15,16]. First notice that one can alternatively reformulate the two constraints ||*β*_1_|| = 1 and ||*β*_2_|| = 1 in Equation (13) to one single constraint ||*β*_1_|| + ||*β*_2_|| = 2 [17]. A possible extension of canonical correlation analysis to *K *time series is then defined by

$$\begin{array}{cc} \text{minimize}\sum_{1\leq k<l\leq K}\sum_{i=1}^{m_{k,l}}L_{c}(\varphi {\left(t_{i}^{\left(k\right)}\right)}^{t}\beta_{k}-\varphi {\left(t_{i}^{\left(l\right)}\right)}^{t}\beta_{l}) & \text{s}.\text{t}.\;\sum_{k\text{=1}}^{K}\left\|\beta_{k}\right\|=K,\beta_{k,j}\geq 0. \end{array}$$

**Figure 5:**
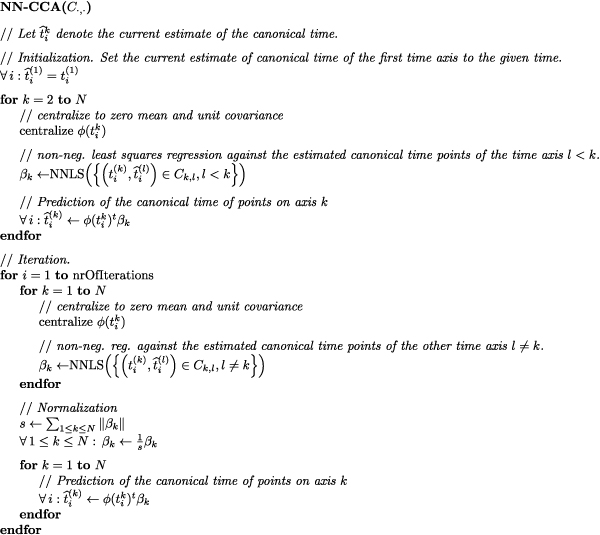
Pseudo-Code for non-negative CCA algorithm.

This problem is again solved iteratively by minimizing the objective function with respect to one vector *β*_*k *_while keeping the other vectors *β*_*l*_, *l *≠ *k *fixed. The pseudo code is given in Figure 5.

## Competing interests

The authors declare that they have no competing interests.

## Authors' contributions

All authors developed the methods, designed and performed comparisons of methods, and wrote the manuscript.
